# Distributed Dynamic Host Configuration Protocol (D2HCP)

**DOI:** 10.3390/s110404438

**Published:** 2011-04-18

**Authors:** Luis Javier García Villalba, Julián García Matesanz, Ana Lucila Sandoval Orozco, José Duván Márquez Díaz

**Affiliations:** 1 Grupo de Análisis, Seguridad y Sistemas (GASS), Departamento de Ingeniería del Software e Inteligencia Artificial (DISIA), Facultad de Informática, Despacho 431, Universidad Complutense de Madrid (UCM), Calle Profesor José García Santesmases s/n, Ciudad Universitaria, 28040 Madrid, Spain; E-Mail: asandoval@fdi.ucm.es; 2 Grupo de Análisis, Seguridad y Sistemas (GASS), Sección Departamental de Sistemas Informáticos y Computación—Lenguajes y Sistemas Informáticos y Ciencias de la Computación e Inteligencia Artificial, Facultad de Ciencias Matemáticas, Despacho 310-F, Universidad Complutense de Madrid (UCM), Plaza de Ciencias, 3, Ciudad Universitaria, 28040 Madrid, Spain; E-Mail: julian@sip.ucm.es; 3 Departamento de Ingeniería de Sistemas, Universidad del Norte, Km. 5 Autopista a Puerto Colombia, Barranquilla, Colombia; E-Mail: jmarquez@uninorte.edu.co

**Keywords:** Mobile *Ad Hoc* Network (MANETs), IP address assignment, autoconfiguration, dynamic host configuration, stateful protocol, synchronization, OLSR proactive routing protocol

## Abstract

Mobile *Ad Hoc* Networks (MANETs) are multihop wireless networks of mobile nodes without any fixed or preexisting infrastructure. The topology of these networks can change randomly due to the unpredictable mobility of nodes and their propagation characteristics. In most networks, including MANETs, each node needs a unique identifier to communicate. This work presents a distributed protocol for dynamic node IP address assignment in MANETs. Nodes of a MANET synchronize from time to time to maintain a record of IP address assignments in the entire network and detect any IP address leaks. The proposed stateful autoconfiguration scheme uses the OLSR proactive routing protocol for synchronization and guarantees unique IP addresses under a variety of network conditions, including message losses and network partitioning. Simulation results show that the protocol incurs low latency and communication overhead for IP address assignment.

## 1. Introduction

A Mobile *Ad hoc* NETwork (MANET) is a set of mobile nodes which communicate through wireless links. In contrast with conventional networks, a MANET does not need a previous infrastructure, since nodes rely on each other to operate themselves, forming what is called multi-hop communication. Such networks have several disadvantages that a conventional network does not present: the topology of this kind of network may change quickly and in an unpredictable way. Moreover, variations in the capacity of nodes and links, and frequent transmission errors and lack of security could occur. Finally, the limited energy resources of the nodes must be taken into account, since normally an *ad hoc* network will be formed by devices powered by batteries.

To communicate with each other [1], the *ad hoc* nodes need to configure their interfaces with local addresses which are valid inside an *ad hoc* network. The *ad hoc* nodes may also need to set routing addresses globally to communicate with other devices on the Internet. From the perspective of the IP layer, an *ad hoc* network is presented as a multi-hop network of level 3 constituted by a collection of links.

In an autonomous *ad hoc* mobile network the nodes can be uniquely identified by an IP address with the only premise that this address must be different from that any other node in the network. The configuration process is the set of steps through which a node obtains its IP address within the network. There are two mechanisms to set addresses: *Stateless* and *Stateful*.

The *Stateless* address configuration proposes its own node to be the one in charge of generating its IP address. The address is obtained from the concatenation of a well-known network prefix and the theoretically unique number inside the network generated by the node. This mechanism may require the inclusion of a module responsible for verifying the uniqueness of the generated address called *Duplicate Address Detection* (DAD) [2–4].

On the other hand, *Stateful* address configuration is based on using servers which control and assign addresses to all the nodes of the network. The well known *Dynamic Host Configuration Protocol* (DHCP) [5] is an example of *Stateful* configuration. However, because of the multi-hop nature of mobile *ad hoc* networks, this protocol cannot be applied directly.

This work proposes a *Stateful*-based auto-configuration protocol that guarantees the uniqueness of IP addresses under a wide variety of network conditions such as missing messages and network partitioning. This work is structured in five sections; the first one is the present Introduction. Section 2 shows the obligated references in the auto-configuration protocol scope of Mobile *Ad Hoc* Networks. Section 3 contains an itemized specification of the so-called *Distributed Dynamic Host Configuration Protocol* (D2HCP), a proposal concerning IP addresses auto-configuration for MANETS. Section 4 presents the D2HCP protocol simulations carried out in NS-3 [6]. Finally, Section 5 discusses the main advantages of the newly developed protocol as well as potential future extensions to the study.

## 2. Related Works

Mobile *Ad Hoc* Networks (MANETS) present special features which must be born in mind when an address configuration protocol is implemented. There are many solutions for conventional networks (e.g., RFCs 3315 [5], 4861 [7], 4862 [8] and so on) but Mobile *Ad Hoc* Networks were not taken into account in their design. It is necessary, therefore, to provide support for multi-hop communication, dynamic topologies and the merging and partitioning of networks, events that are typical in Mobile *Ad Hoc* Networks.

There are numerous works that present proposals for address configuration in a Mobile *Ad Hoc* Network using the *Stateless* and *Stateful* mechanism. Without doubt, the most representative are those described in [2,9–21]. Bernardos *et al.* [22–24] carried out a rigorous study of the problems of the auto-configuration in Mobile *Ad Hoc* Networks, presenting an itemized review of the more representative auto-configuration protocols. A comprehensive review of the main auto-configuration protocols can be found in [25].

The *Internet Engineering Task Force* (IETF) [26] includes what is perhaps the best known work group in this field, the so-called *Ad Hoc Network Autoconfiguration Work Group* (Autoconf WG) [1] whose principal purpose is to describe the addressing model for *ad hoc* networks and how the nodes can set their addresses in these networks. It is essential that such models do not cause problems to other components of an *ad hoc* system such as standard applications which are executed in an *ad hoc* node or Internet nodes connected to the *ad hoc* nodes. The work of this group can include the development of new protocols if the existing IP auto-configuration mechanisms turn out to be inadequate. Nevertheless, the first task of this work group is to describe a practical addressing model for *ad hoc* networks.

The solutions described previously represent significant contributions to aid our comprehension of the problem, but we consider that all these approaches only handle a subset of the network conditions enumerated as follows:

1. Dynamic Topology: the nodes in the network can move arbitrarily and may join and leave the network dynamically.
2. Message loss and failure in the nodes: message loss can be quite frequent and can result in duplicate IP address allocation if it is not managed correctly. The nodes can abruptly depart from the network due to a link failure or an accident.
3. *Partitioning* and *merging*: the network can split into multiple networks and, later, join with others. During network merging it is possible to have duplicated IP addresses in the fused network.
4. Address concurrent requests: multiple nodes may want to join the network simultaneously.
5. Limited Energy and Bandwidth: the nodes in a Mobile *Ad Hoc* Network have limited energy and the links have a limited bandwidth, therefore, the communication overhead which is incurred should be low.

In this work a solution similar to DAAP [27,28] and to the one given in [29] that guarantees uniqueness in the IP address allocation under a wide set of network conditions is proposed. In our approach, the majority of address allocations imply local communication, thus causing low communication overhead and low latency.

## 3. D2HCP (Distributed Dynamic Host Configuration Protocol)

The Distributed Dynamic Host Configuration Protocol (D2HCP) is an auto-configuration protocol that manages the addition and departure of nodes in a MANET. The protocol makes the MANET nodes collaborate with each other to manage the assignment of unique and correct IP addresses in a distributed manner. All the network nodes have the same role; there is no special type of node that centralizes the management of the same.

Nodes have a synchronization system based on the OLSR [30] routing protocol. Thanks to this mechanism, the synchronization is done passively, by monitoring the mentioned routing protocol, thus no network traffic overhead is generated compared to that generated by the OLSR protocol.

Due to the fact that all the nodes are responsible for managing the addition of any new node to the network, this process can be done quickly. A node that wishes to join a network tries to contact any node still belonging to it, and may receive several responses from multiple nodes. This makes the chances of successfully joining the network high, because of the high availability and redundancy that distributed management provides.

Here we introduce the D2HCP specification: it begins with the data structures used, continues with an explanation of the messages exchanged between nodes for joining and departing the network, and then details how synchronization takes place in the protocol. Finally, we explain the format of the messages exchanged during the auto-configuration process, detailing how to solve the problem of possible message loss in the network using appropriate timers and performing certain actions when they expire to restore the auto-configuration process, as well as state diagrams for each operation mode that a node can adopt.

### 3.1. Data Structures

The data structures of this protocol can be classified into those handling the auto-configuration mechanism and those belonging to the OLSR routing protocol. OLSR stores internally a routing table which is updated periodically. This table contains information about the route to each node, stored in the following fields:

- *R_dest_addr*: IP address of the destination node.
- *R_next_addr*: IP address of next hop in the route.
- *R_dist*: Distance to the destination node.
- *R_iface_addr*: IP address of the outgoing interface to the destination node.

The structures necessary for auto-configuration mechanism are:

- IP addresses of the node interfaces.
- Netmask.
- Free_IP_Blocks: A table of free block from each node in the network. It will have the following form:

**Table:** 

**IP**	**Free_IP_blocks**
.1	.1–.64
.128	.128–.254
.65	.65–.127

### 3.2. Joining and Departure of Nodes

The protocol uses a specific message number for each operation. All the operations are defined seeking optimum operation and low latency. This section discusses how communication is established between the nodes and the messages transmitted during the joining and departure of nodes in the network.

#### 3.2.1. Node Joining

**Figure 1.**
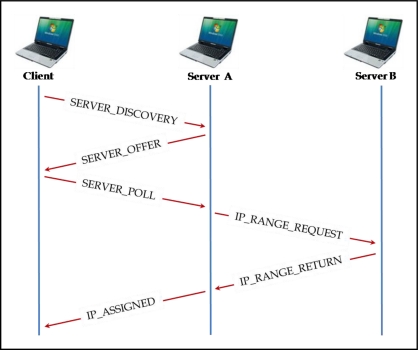
The messages interchanged in the process of a node joining the network.

The entry of a node to the network implies the need to locate a node acting as a server. Once found, it will facilitate the joining by providing an IP address block and a *Free_IP_Blocks* table representing the state of all the nodes in the network. Until the node has an assigned IP address, its communication with nodes which might act as servers will be through the MAC layer. The configuration mechanism uses four types of messages in most cases. If no nodes in range with free IP addresses are found six types of messages in total will be used. Figure 1 shows the exchanged message scheme as is explained below:

1. *SERVER_DISCOVERY:* The client node wishing to join a network starts the process with a message of this type. It is transmitted by the MAC layer, with the *broadcast* address as its destination. The message indicates the IP address number which is required (equal to the interface number). If the node has more than one network interface, the message is transmitted through all of them, using the ID field, thus the different interfaces are not *confused* with several nodes.
2. *SERVER_OFFER:* The network nodes receiving the SERVER_DISCOVERY message reply to this message, also using the MAC layer, in which an IP address number is offered. The number of addresses offered is half of the available range. The SERVER_DISCOVERY message includes a *Count* field indicating how many attempts have been made by the client. Depending on its value, the server nodes will behave as follows:
  - *Count* = 1: The server node will respond with a SERVER_OFFER if enough addresses are available and the fields R (*Ready*) and L (*Local*) have the value 1 (it can assign the addresses provided in the moment, and they are addresses from the node’s own block).
  - *Count* = 2: The node server will respond with a SERVER_OFFER if the fields can take the value R = 1 and L = 1. If not possible, it will still also respond if it is the case that there are enough addresses and R = 0, L = 1 (the server cannot assign addresses at the moment, but it has them).
  - *Count* > 2: If the node has addresses available and is in a state to do so, it will send a SERVER_OFFER with R = 1, L = 1. If it can, will send it with R = 0, L = 1. Finally, if it does not have enough free addresses, it will send the message with the fields R = 1, L = 0 (immediate availability of addresses, but the offered addresses are from another node in the network).
3. *SERVER_POLL:* After a certain listening time, the client node will have received several SERVER_OFFER messages. If not, it will try again. It will sort received messages using the following criteria:
  - The servers which are not available are discarded, *i.e.*, with R = 0. The SERVER_OFFER with R = 0 is not used to reply with a SERVER_POLL, but they have the function of informing the client that there is a server node in the network, although it cannot provide access to it at this moment.
  - Priority is given to local addresses: it will prefer messages with the field L = 1.
  - Finally, it is organized so that the offered addresses are ranked, from highest to lowest.
  According to this criteria for order preference, it will send a SERVER_POLL message to the first server (via the MAC layer, again) to let it know that the node has chosen this one to assign a free IP address block to it.
4. *IP_RANGE_REQUEST:* If the addresses provided by the server node were not their own, but they were from a third node in the network, with this message there will be a formal request made to that node. Since there is communication between two nodes already configured correctly, it is performed at the IP layer.
5. *IP_RANGE_RETURN:* The third network node authorizes the node that sends the message IP_RANGE_REQUEST to assign the address block indicated in this message to client nodes. It is also a message sent by IP.
6. *IP_ASSIGNED:* After receiving the SERVER_POLL, if the provided addresses were from the server node’s own ones, or after an IP_RANGE_RETURN message if it was necessary to request the address from a third node, the node server sends this message to the client. This message is transmitted by the MAC layer. In this message the free address block which is assigned to the client and *Free_IP_Blocks* table representing the network state are indicated. The table which is transmitted in this message does not reflect the joining of the client node.

After this message exchange, the client node chooses the first one of the block which has been assigned as its IP address. In the case of having more than one network interface, it will use the first ones of block in order, and will be the first of all which use as the primary address that identify the node.

#### 3.2.2. Node Departure

The node departure mechanism does not require the exchange of any messages. The node that wants to leave the network does not have to notify any other node of its departure, avoiding the overhead that these messages cause. The other nodes in the network will become aware of the departure node through the periodic route updates that the OLSR protocol performs every so often. They will note that they have lost the path to that node, and therefore they remove it from their *Free_IP_Blocks* table, adding its free address block to the corresponding node as explained in the previous section.

### 3.3. Synchronization

The synchronization is done by monitoring the routing table of the OLSR routing protocol [30]. The addition or departure of a node in the network is detected when OLSR adds a new route to its routing table, or deletes an existing one. By detecting the addition or departure of a node in the network, the *Free_IP_Blocks* table is updated locally, and without exchanging any messages.

For this reason, the following rules are obeyed:

- The responsibility of recovering the IP addresses that a node leaving the network makes available is one that can be attached to the right of the free block. This will not be possible when the block to be collected contains the lowest address of the network. In that case, the node that picks the block up is one that can add to it to the left.
- By dividing the free addresses in two blocks to deliver one of them to a new node that joins the network, the node that acts as server delivers the sub-block, which does not contain its own IP address, to the client.

When the node departure is detected, its entry must be removed, and an update of the corresponding node’s now available IP address must be recorded.

By detecting the joining of a new node, a new entry is created for it in the table, and the free address block of the node which supplied its IP address will be updated. In order to identify the node who acted as a server, it is simply necessary to find out which node has the IP address of the new node in its free address block.

### 3.4. Message Format

**Figure 2.**
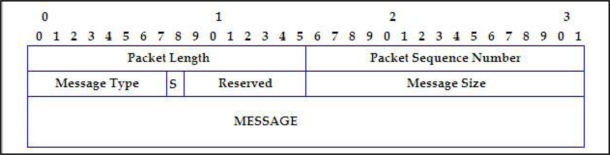
Packet from the D2HCP protocol.

All the sent messages are packed in the protocol with the format shown in Figure 2.

These messages will be encapsulated in turn with the headers corresponding to the MAC or TCP/IP, depending on the type of message to be included in the MESSAGE field.

#### 3.4.1. Packet Header

The first row of the Figure 2 contains the fields of the packet header.

1. *Packet Length*: Packet length, including the header (2 bytes).
2. *Packet Sequence Number*: Sequence Number (2 bytes). In each different message which is sent by node, this field is increased by one. It helps in being able to detect duplicated packets.

#### 3.4.2. Message Header

The second row of the Figure 2 is the header of each one of the protocol message:

1. *Message Type*: It has 1 byte of size.
2. *S (Security)*: Reserved for security implementation (1 bit).
3. *Reserved*: Reserved for functionality future extensions (7 bits).
4. *Message Size*: It consists of 2 bytes.

Next the format of each kind of message include in the MESSAGE field is shown.

- SERVER_DISCOVERY

**Figure 3.**
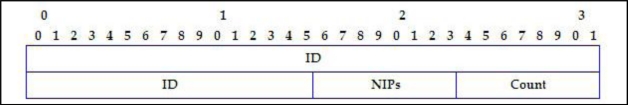
SERVER_DISCOVERY message format.

Figure 3 shows the SERVER_DISCOVERY message format.

*ID*: Node Identification (6 bytes).The node has to choose the MAC address from one of its interfaces, if it has more than one. This identity field has the same value for every SERVER_DISCOVERY and SERVER_POLL message emitted by the node, although it was doing from different interfaces.

1. *NIPs*: Amount of IP addresses solicited by the node. It will be equal to the interface number of client node (1 byte).
2. *Count*: Number of times that the SERVER_DISCOVERY petition has been tried (1 byte).

- SERVER_OFFER

**Figure 4.**
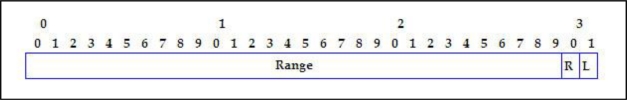
SERVER_OFFER message format.

Figure 4 shows the SERVER_OFFER message format.

1. *Range*: Number of IP addresses offered (30 bits).
2. *Ready* (R): It indicates whether the node that offers the IP addresses is ready to assign them or only communicates their existence but at this point cannot assign them (1 bit).
3. *Local* (L): It indicates whether the range offered is from the sending node, or else be asked to turn to a third node (1 bit).

- SERVER_POLL

**Figure 5.**
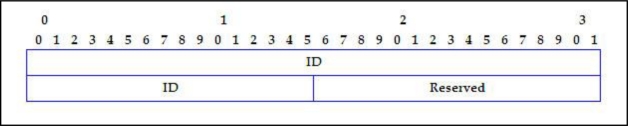
SERVER_POLL message format.

Figure 5 shows the SERVER_POLL message format.

1. *ID*: Node identification (6 bytes). It is the same identification as that elected in the SERVER_DISCOVERY message.
2. *Reserved*: Reserved for future implementations (2 bytes).

- IP_ASSIGNED

**Figure 6.**
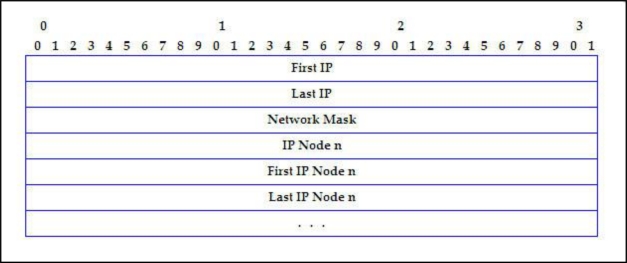
IP_ASSIGNED message format.

Figure 6 shows the IP_ASSIGNED message format.

1. *First IP*: IP address start of a free address block (4 bytes).
2. *Last IP*: IP address end of free address block (4 bytes).
3. *Network Mask*: It consists of 4 bytes.
4. *IP Node n, First IP Node n, Last IP Node n*: This represents an entry on the free block table for all the nodes in the network. Each node is represented by these fields of 4 bytes, each one being: the node IP address, the initial IP address and the final from its free address block, respectively.

- IP_RANGE_REQUEST

**Figure 7.**
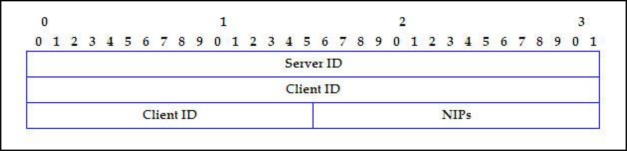
IP_RANGE_REQUEST message format.

Figure 7 shows the IP_RANGE_REQUEST message format.

1. *Server IP*: Server address that requests the range for client (4 bytes). It can be different from the IP address of the sender message, by treating multi-hop networks.
2. *Client ID*: Client node identification. It has the same value as the ID field of the SERVER_DISCOVERY and SERVER_POLL message (6 bytes).
3. *NIPs*: Requested IP Address Number (2 bytes).

- IP_RANGE_RETURN

**Figure 8.**
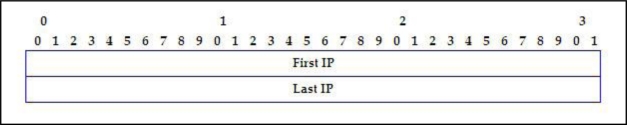
IP_RANGE_RETURN message format.

Figure 8 shows the IP_RANGE_RETURN message format.

1. *First IP*: The initial IP address of the free address block (4 bytes).
2. *Last IP*: The final IP address of the free address block (4 bytes).

### 3.5. Timers

The wireless and mobile nature of MANET means that there are situations in the networks where messages are lost, or delayed in getting to its destination more than the estimated time. Therefore, we expose below a series of timers used to solve such situations:

- *SERVER_DISCOVERY_TIMER*: After sending the message, the client node will start this timer when it gets to the state WAITING_REPLY. During this time the node is waiting for SERVER_OFFER messaged of possible close by nodes belonging to a network.

The longer the timer runs, the more time it will dedicate to receiving messages of this kind, thus there will be more to process and, therefore, it is easier to get an address block. But it also means increasing the latency to obtain an IP address.

If this timer expires and the client node has not received any SERVER_OFFER, there has been a message loss, or perhaps there are no server nodes, it will send a new SERVER_DISCOVERY. This action is repeated a maximum number of times (SDISCOVERY_MAX_RETRY) and, if it goes on without receiving messages, it will initiate its own network.

- *SERVER_OFFER_TIMER*: After the SERVER_OFFER message, the node goes into the WAITING_POLL state, and starts this timer. When it expires, the state will change to IDLE.
- *SERVER_POLL_TIMER*: As soon as the SERVER_POLL message is sent, the node client will wait for the IP_ASSIGNED message for the time that this timer should determine. If this message does not come, the SERVER_POLL will be re-transmitted up to a maximum number of attempts, defined as SPOLL_MAX_RETRY. If the maximum number of attempts is exceeded, it will begin the configuration process again.
- *IP_RANGE_REQUEST_TIMER*: The server node who sends an IP_RANGE_REQUEST message to another node of the network initiates this timer at that moment. As with the SERVER_POLL_TIMER, if this timer expires, the IP_RANGE_REQUEST message will be forwarded up to a maximum number of attempts, given by RREQUEST _MAX_RETRY.
- *ACCEPTED_OFFER_TIMER*: This timer is activated after sending an IP_ASSIGNED message or an IP_RANGE_RETURN message. During this time, the server node cannot reply to requests of SERVER_POLL or IP_RANGE_REQUEST type. This restriction will arise after the timer expires (the offer expired without being accepted), or on having detected that a node with the first IP address of the offered ones has entered the network (the offered address block was accepted). It is necessary to bear in mind that although it could not assign IP address, the server node will keep on answering SERVER_DISCOVERY requests giving the value 0 to the field R (READY) in the SERVER_OFFER message. In this way, the node client is informed of the existence of the server, although it should not be capable of assigning IP address immediately.
- *NODE_DOWN_TIMER*: When OLSR erases the route towards a node, it is not eliminated immediately from the *Free_IP_Blocks* table. In its place, this timer is initiated. If before the timer expires it manages to discover a route to the node, that means that it disappeared momentarily, but it did not leave the network. Therefore, the elimination is cancelled on the *Free_IP_Blocks* table. In case the timer expires and a route has not been recovered, the node is assumed to be lost and its entry is eliminated from the *Free_IP_Blocks* table, updating those who match.
- *INIT_TABLE_TIMER*: On receiving the *Free_IP_Blocks* auto-configuration table in the IP_ASSIGNED message, the client node activates this timer. During that time, the table contains nodes so that OLSR does not have a well-known route yet. After the timer expires, the nodes from the *Free_IP_Blocks* table that do not have entries in the OLSR route table are verified: these nodes are eliminated (updating the corresponding entries), since they are nodes that belonged to the network on having received the table, and have left it before OLSR knew of its existence.
- *INIT_ASSIGN_TIMER*: This timer is used as much by the node client as by the server when they have received or assigned an IP address block, respectively. That is to say, the server initiates it, on having verified the node entry with the first IP address of the block offered in the IP_ASSIGNED message, and the client initiates it after receiving the IP_ASSIGNED message and to configure its address. Thus there has been time for the whole network to update its Free_IP_Blocks table before more changes take place. During that time, they will ignore SERVER_POLL or IP_RANGE_REQUEST messages, although they will reply to the SERVER_DISCOVERY.
- *NODE_DOWN_ASSIGN_TIMER*: When an already configured node detects the departure of another one, and it verifies that it is its turn to gather the IP address that remains free, it starts its timer. More concretely, the timer will be activated when the elimination of the OLSR routing table is detected, that is to say, it will be activated at the same time as the NODE_DOWN_TIMER timer.

Until it does not expire, the node will ignore the SERVER_POLL and IP_RANGE_REQ requests. This way, a margin of time will happen to ensure that all the nodes in the network detect the mentioned departure and update their *Free_IP_Blocks* table, before assigning them to some another new node. Therefore, the duration of this timer must be greater than that of the NODE_DOWN_TIMER to make sure that the rest of nodes in the network not only detect the elimination of a route but they have eliminated it from the *Free_IP_Blocks* table. The node will keep on replying to the SERVER_DISCOVERY messages fixing the value 0 in the R field from the SERVER_OFFER message.

- *SLEEP_TIMER*: Timer used by a client node when it detects nearby nodes belonging to some network, but that are not in a position to assign IP addresses at this moment. This way, it gives a margin of time to allow the processes to end preventing them from assigning address.

### 3.6. State Diagrams

Depending on whether they are in the process of joining the network, or if they already belong to one, two types of nodes are differentiated: client and server. In the following paragraphs the state diagrams that govern the behavior of both types of nodes are shown and explained. The state, in which the node is found, changes when sending or receptions of messages ocurrs, or when determined timers expire.

#### 3.6.1. Server Node

**Figure 9.**
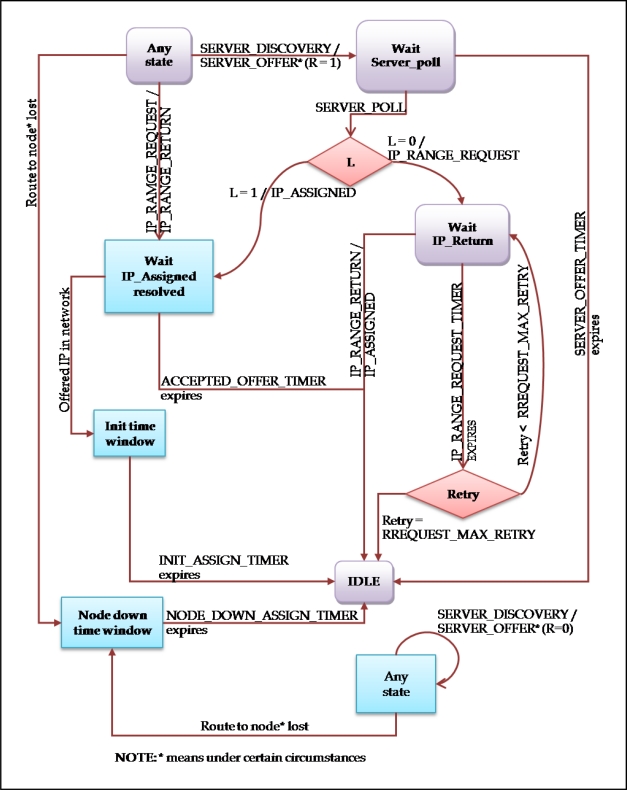
Server Node State Diagram.

We call all the nodes in the network that are configured correctly server nodes, that is to say, they possess a valid IP address with which they can communicate with the rest of nodes, and a free IP address block. With this free address block they facilitate access to the new nodes, which we will call clients. Figure 9 shows the state diagram. As we can see, two types of states exist: the ones represented with rounded and clear rectangles, and those enclosed in somewhat darker rectangles with corners. We will call them states of the type *ready* or *not_ready*, respectively.

From any state, the server node is in expectation of SERVER_DISCOVERY messages. It will always reply with a SERVER_OFFER message, but depending on whether the current state of the node is of *ready* or *not_ready* type, it will answer giving to the field R (READY) the value 1 or 0. This way, a node always announces its presence, although at this precise moment it should not be capable of assigning IP addresses to a client. This is indicated in the state diagram with the any state state.

- *Any state (ready):* From any state of ready type, the server node will respond to a SERVER_DISCOVERY message with one of SERVER_OFFER type (R field with value 1). That will ensure that the server goes to the state Wait SERVER_POLL. A node can reply SERVER_DISCOVERY at the same time to requests from different nodes, and be awaiting any of the corresponding SERVER_POLL messages. Also they will answer messages of type IP_RANGE_REQUEST with one of the type IP_RANGE_RETURN. This means that whether the node is in any ready state, it will be give half of its free address blocks to any other node in the network without proper addresses and that it needs to facilitate the joining to a client.
- *Any state (not_ready):* Whilst the node is in a *not_ready* state, it will reply to the SERVER_DISCOVERY messages with a SERVER_OFFER message giving the value 0 to the field R.
- *Wait SERVER_POLL:* In this state the node waits for a time determined by the SERVER_OFFER_TIMER timer to receive a SERVER_POLL message. This message indicates that the client, the one who sent the SERVER_OFFER has chosen as its server for the process of auto-configuration. After the reception of the message, the node will send a IP_ASSIGNED message to the client in the case of having available addresses locally (the field L of the message SERVER_OFFER had value 1); and it will pass to the Wait IP_Assigned state resolve. If the addresses that it offered were not local, it will have to ask for them from a node in the network with the IP_RANGE_REQUEST message; and it will pass into the state Wait IP_RReturn. In case any SERVER_OFFER messages are not received before the timer expires, the node will pass to be in the IDLE state.
- *Wait IP_ASSIGNED resolved:* In this state, of type not_ready, the node is waiting to find out if the client node correctly received the address block offered by an IP_ASSIGNED message, or through an IP_RANGE_RETURN message and an intermediary. The result can be that the client has been configured, or that it has not received the address block. The above-mentioned can take place for several reasons, since they can be problems with interferences in the message reception, movement of the client node out of the coverage range, and so on. If a new node appears in the network using the first IP address from the block offered in the message IP_ASSIGNED or IP_RANGE_RETURN, it means that the client node stopped being configured. In this moment the server node changes its state *to Init steal window*. If the node was not capable of finishing the auto-configuration process, the ACCEPTED_OFFER_TIMER timer will expire. In this case the node passes to the IDLE state.
- *Init time window:* This state serves to give a time margin that allows all the nodes in the network to be capable of detecting the joining of the recently configured client, before dividing again their own IP free address blocks. If this margin did not exist, and new requests would be attended immediately, synchronization problems might happen if other nodes were detecting the new incorporations to the network in an incorrect order.
- *Wait IP_return:* In this state the node is waiting for the reception of an IP_RANGE_RETURN message. When it receives this message, in which a node in the network indicates it has a block that it can offer the client in waiting; it will send an IP_ASSIGNED message to the client. After this, the node will have ended its function as server, and will pass to the IDLE state. If the IP_RANGE_RETURN message is not received before the timer IP_RANGE_REQUEST_TIMER expires, it will turn to try the request sending again an IP_RANGE_REQUEST message a maximum number of times RREQUEST_MAX_RETRY. These successive attempts are sent in every occasion to a different node. If the limit of attempts is exceeded, then the node will desist and change its state to IDLE.
- *IDLE:* This is the state of rest, or the one in which the node is idle. When it is in this state, the node does not undergo any operation related to the auto-configuration process. It is therefore treated as a waiting state.
- *Node down time window:* This node provides a time margin when the node must gather the IP address from a node that has left the network. More precisely, the node changes to this state on having detected that it has lost the route towards a node of whose address block it is responsible. This transition is done from any other state, be it of type ready or not_ready. After the time determined by the timer NODE_DOWN_ASSIGN_TIMER, the node will return to the state of rest IDLE. This timer is not to be confused with the NODE_DOWN_TIMER. Although they begin at the same time on having detected the same event, the processes involved are independent.

#### 3.6.2. Client Node

**Figure 10.**
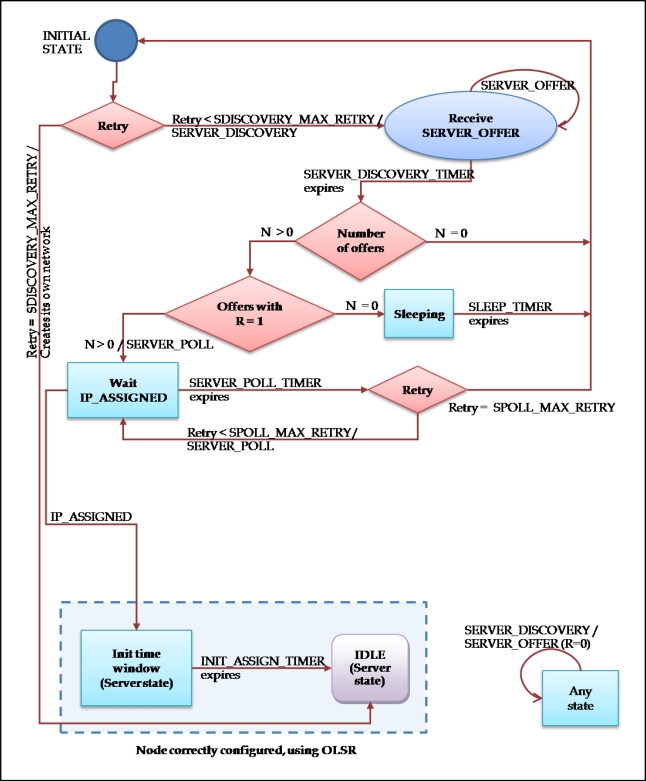
Client Node State Diagram.

The procedure that a node that wants to gain access to a network follows is described in Figure 10.

The diagram is provided with *not_ready* type states, explained in the state diagram of the server node and represented with the same style of rectangles. As if it were an already configured node, the node that is in process of configuration of its IP address replies to SERVER_DISCOVERY requests with SERVER_OFFER messages. In these messages the value 0 is given to the R field, since the node is not in a position to assign IP addresses, and only tries to announce its presence.

- *Initial state:* in which the auto-configuration process begins. If the number of attempts is less than the maximum, SDISCOVERY_MAX_RETRY, then the node emits a SERVER_DISCOVERY message for each of its network interfaces which are going to use the MANET network. It changes its state to Receive SERVER_OFFER. If it has gone over at the limit of attempts, then the node desists from his intention from finding a network to join and creates a new one. It will become a node server, beginning in the IDLE state of the server state diagram.
- *Receive SERVER_OFFER:* It is treated as a state of waiting, during which the SERVER_OFFER messages of other possible nodes are gathered. On having ended the waiting period, determined by the timer SERVER_DISCOVERY_TIMER, the responses are processed. If no SERVER_OFFER response has been received, it is returned to the initial state. If there is some offers, the number of them with the value 1 in the field R is verified. If among the offers none had the bit R set to 1, it means that there are nearby nodes belonging to a network, but at the moment they are not capable of assigning IP addresses. Therefore, the node passes to the Sleeping state.

If there was some offer with the bit R set to 1, the servers are sorted by preference and a SERVER_POLL message is sent to the first one of them. In this case the node changes its state to Wait IP_ASSIGNED. This state is not one of the types explained in the state diagram of a client node. This means that it does not reply to SERVER_DISCOVERY messages, since at the moment it does not know if there is any network nearby which to join.

- *Sleeping:* The node interrupts its attempts to join the network during the time determined by the SLEEP_TIMER timer. This is like that because there have been received SERVER_OFFER messages of nearby nodes that at the moment are not capable of assigning IP addresses, and what is claimed that after this time they are already capable of facilitating the join to the network. After the timer expires, the node will return to the *initial state.*
- *Wait IP_ASSIGNED:* In this state, the client is in expectation of an IP_ASSIGNED message on the part of the server to whom the message SERVER_POLL was sent. If the awaited message does not come, the SERVER_POLL_TIMER timer expires. In this case, it will turn to try to send a SERVER_POLL to the following server of the list generated after the end of the SERVER_DISCOVERY_TIMER timer. If the list of servers is ended, or it goes over the limit of attempts SPOLL_MAX_RETRY, the node returns to the initial state. On having received the IP_ASSIGNED message, the node configures its address (or addresses, in case of having several network interfaces). At this moment, it already takes part normally in the network, and passes to be a node server. The state with the one begins its behaviour as server is the *Init time window.*

## 4. Simulations and Results

Since in these networks nodes numbers that form the network are unpredictable, the protocol scalability is one of the main issues to consider. Therefore, it is essential to evaluate the impact of increasing the nodes number in the network in distinct parameters such as latency in address assignment, the overhead because of control traffic or delay in the synchronization. Besides the nodes number in the network, it is necessary to take into account the frequency of the input and output nodes in the network. When a node leaves the network, the free address tables free in the network have been updated. If this is not done quickly, the network nodes cannot deal with requests for new entries in *the network to int*erpret that the network does not have free addresses.

To evaluate the D2HCP protocol performance has been used Network Simulator Network Simulator 3 (NS-3) [6]. Different scenarios of MANET networks were simulated to evaluate performance under different circumstances.

### 4.1. Simulation Scenarios

Table 1 summarizes the main parameters used during simulations. When performing these simulations has been remained constant the entries number in the network per unit time. This factor is important, particularly in high density networks.

### 4.2. Results

**Figure 11.**
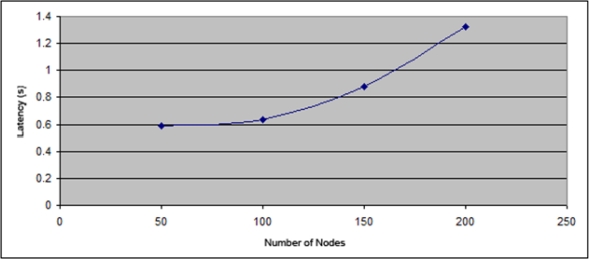
Latency in the IPv4 address assignment of a network from Class C.

**Figure 12.**
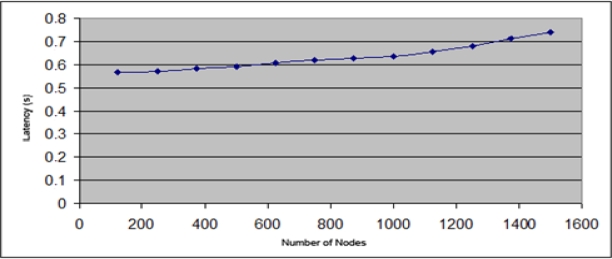
Latency in the IPv4 address assignment of a network from Class B.

Firstly the latency in the process of assigning addresses. Figures 11 and 12 show the evolution of the latency value depending on the nodes number in the network. Figure 11 shows the values using IPv4 addresses from class C, *i.e*., with 254 available addresses. Figure 12 uses IPv4 addresses from Class B, providing 65534 addresses. Figure 11 shows that the increase in time address allocation begins to grow more quickly from the 125 nodes. However, using addresses from class B (Figure 12), the time experiences a very slight growth up to 1,600 nodes which were simulated.

These results indicate that the parameter that further determines the latency is the percentage of occupied addresses. When this percentage is nearly 50% the node number that do not have addresses to offer increases in direct proportion way. This does not allow a local assignment to be done and it is necessary to request the address from another node, increasing the time needed to complete the process. Anyway the average latency in the address assignment process is low.

Against auto-configuration protocols based on auto duplicate address detection (DAD), the protocol D2HCP also presents a great reduction in the overhead of control packets in the network.

In fact, in most cases, the configuration is performed locally, *i.e.*, a neighbor will assign address to the new node. This involves the sending of four control packets which do not spread to the rest of the network. In the case where no local address may be assigned, a *unicast* transmission is performed with the chosen server, which causes much less overhead than a *broadcast* sending. The probability that cannot be assigned addresses locally depends on the relationship between the node number in the network and the available address number.

**Figure 13.**
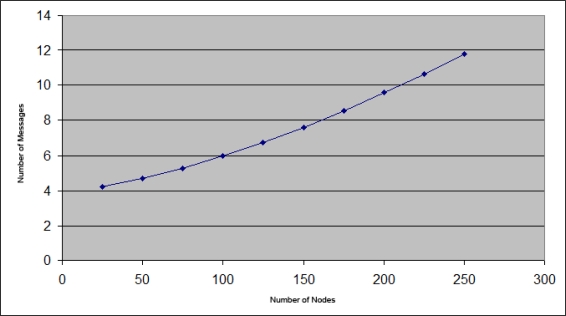
Control message average number sent in each address configuration.

In Figure 13 we can see the average number of control packets involved in each address configuration process. In the simulations have been used IP addresses from Class C, thus we have 254 network addresses. In Figure 13, you can see that when there are few nodes in the network, the required control message number to carry out auto-configuration is near the minimum, since in most cases the configuration can be performed locally.

However, when the free address number is close to 0, no configuration can be performed locally and remote nodes must use to perform such configuration, increasing the sent message number.

**Figure 14.**
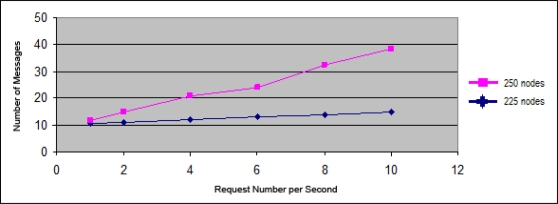
Control message number against to number of requests per second.

Figure 14 shows the evolution of the necessary control message number to assign an address based on the request number per second. In the case of the pink line the simulation was carried out in a scenario with 250 nodes. In the case of the blue line a scenario with similar characteristics has been used, but with 225 nodes. The used frequency of node departures in the network is similar to the frequency of node joins to hold the address availability.

As shown in Figure 14 in the case of a network with 225 nodes (about 90% occupancy) the required control messages number is practically independent from the request number per second, which means that the protocol efficiently supports the network scalability.

Only in the limit (around 100% occupancy) the protocol reduces its performance in terms of overhead. In fact, the main performance problem found is given in the situation that reflects the pink line in Figure 14. In situations where errors occur in the choice of the remote server, the latency increases in direct proportion to the control message number sent. However, this increase in overhead is acceptable, since it is still lower than the overhead incurred by the DAD algorithms.

### 4.3. D2HCP *versus* the Thoppian and Prakash Protocol

**Figure 15.**
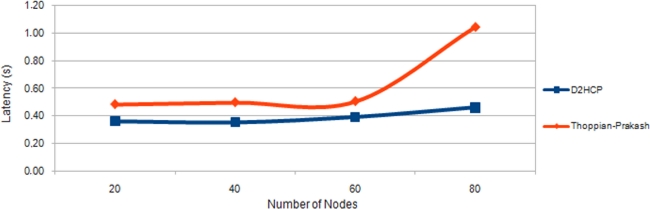
Latency D2HCP *versus* Thoppian and Prakash Protocol.

Figure 15 shows a comparison of the latency of D2HCP *versus* the Thoppian and Prakash Protocol. It is noted that in the first case the latency is lower than the second and it is very regular too (in Thoppian and Prakash the latency grows exponentially when the number of nodes is high) allowing us to conclude that D2HCP improves the results of its predecessor.

## 5. Conclusions and Future Work

An auto-configuration protocol for Mobile *Ad Hoc* Networks called D2HCP *Distributed Dynamic Host Configuration Protocol* (D2HCP) has been designed. This protocol is classified as a *stateful* protocol. This is an IPv4 address auto-configuration protocol for isolated Mobile *Ad Hoc* Networks.

In this protocol each node is responsible for managing a range of addresses. When a new node wants to begin participating in the network, one of the nodes within the network gives half of its address range to the new node. In the case of any adjacent node not having free addresses, but free addresses do exists, a request to a network node that has free addresses is done. In this operation mode is based on distributed nature of the protocol.

To keep updated information about free addresses owned by each node, the traffic of control packets from OLSR protocol. Such protocol at each node tries to keep updated knowledge of the whole topology from the network. This protocol has been designed to work together with OLSR; although it could operate with any proactive protocol by the flexibility of its design.

D2HCP warrants uniqueness for IP addresses in a wide variety of network conditions including message loss, concurrent requests and network partition. The simulation results show that the protocol has low latency and overhead. Worth noting is the protocol scalability features compared to other proposals in the literature, its flexibility that facilitates the protocol extension with new features, as well as synchronization process introduces null overhead.

Possible future work can be identified as follows:

- Detection of the *merging* to allow reassigning addresses that enters in conflict (something relatively easy since it would introduce a new message).
- Extension of the protocol to subordinate networks with access to the Internet or other networks, for which it should take into account the network topology to perform address auto-configuration process.
- Study protocol performance in cooperation with other proactive routing protocols (the first version D2HCP is designed to work together with OLSR).
- Add a security module that protects against different attackers to proportionate a safe auto-configuration.

## Figures and Tables

**Table 1.** Simulation Parameters.

**Parameter**	**Value**
Simulation Area	1,500 m × 1,500 m
Mobile Node Number	50 to 1,600
Mobility Pattern	Random Waypoint (*setdest*)
Routing Protocol	OLSR
Node Range or Coverage	125 m
Simulation Number	10
Simulation Area	1,500 m × 1,500 m
